# Transcriptomics and Plant Hormone Analysis Reveal the Mechanism of Branching Angle Formation in Tea Plants (*Camellia sinensis*)

**DOI:** 10.3390/ijms26020604

**Published:** 2025-01-13

**Authors:** Jinping Zhu, Xiaoman Li, Jianyan Huang, Lu Wang, Qinghua Zheng, Hanjia Li, Yao Chen, Junwei Tang, Xinyuan Hao, Xinchao Wang, Youyi Huang, Jianming Zeng

**Affiliations:** 1 National Key Laboratory for Germplasm Innovation & Utilization of Horticultural Crops, Tea Science Department, College of Horticulture and Forestry, Huazhong Agricultural University, Wuhan 430070, China; z2663276245@163.com; 2 National Centre for Tea Plant Improvement, Tea Research Institute, Chinese Academy of Agricultural Sciences/Key Laboratory of Biology, Genetics and Breeding of Special Economic Animals and Plants, Ministry of Agriculture and Rural Affairs, Hangzhou 310008, China; lixiaoman@tricaas.com (X.L.); huangjianyan@caas.cn (J.H.); wanglu317@tricaas.com (L.W.); zqh3350485471@163.com (Q.Z.); lhj1053535490@163.com (H.L.); chenyao@tricaas.com (Y.C.); tangjw6140@163.com (J.T.); haoxy@tricaas.com (X.H.); xcw75@tricaas.com (X.W.)

**Keywords:** plant hormones, transcriptome analysis, branching angle, tea plant (*Camellia sinensis*), lateral bud

## Abstract

The branching angle of tea plants is a key factor in determining their branching structure, which significantly affects yield, suitability for mechanical harvesting, and overall plant architecture. However, the mechanisms underlying branching angle formation in tea plants remain unclear. In this study, we explored the mechanism of branching angle formation in tea plants by analysing the transcriptome and plant hormone levels of tea plant cultivars with different branching angles. The results indicated that gibberellin positively regulates the branching angle of tea plants, cytokinins, auxin, and abscisic acid involved in the formation of branching angles in tea plants. The transcriptome analysis revealed that candidate regulatory factors, including plant-hormone-related genes (the gibberellin synthesis gene *GA3ox1* and metabolism gene *GA2ox1*; the cytokinin metabolism genes *CKX* and *UGT*; the auxin signal transduction-related gene *LAX3*; and the abscisic acid signal transduction gene *PYL4*), genes regulating cell division and growth (*LAZY1*, *TAC1*, and *MAX1*), and transcription factors (MYBs, WRKYs, TCPs, AP2/ERFs, and MADS-box), are involved in the formation of branching angles in tea plants. These results offer insights into the mechanism of branching angle formation in tea plants, providing important theoretical reference for the selection and breeding of tea cultivars suitable for mechanical harvesting, thereby improving agricultural efficiency and sustainability.

## 1. Introduction

Branching angle is a key component of plant architecture, which directly affects crop yield, quality, and stress resistance [1]. While crop height and tillering have been extensively studied, the mechanisms underlying branching angle formation remain relatively unexplored [2]. Crops with wide branching angles tend to have dispersed crowns, which can hinder mechanical operations [3]. In contrast, smaller branching angles contribute to a denser and more compact plant structure, promoting higher yield [4]. Lateral bud growth plays a significant role in determining the branching angle, which is crucial for shaping the overall plant architecture [5].

The formation of branching angles is regulated by genetic, environmental, plant hormonal, and nutrition factors [6]. Cytokinins (CKs), synthesised in the root and distributed throughout the plant, influence lateral bud growth and angle formation in *Arabidopsis* through *CYTOKININ OXIDASE/DEHYDROGENASE (CKX)* genes [7]. In rice, overexpression of *CKX3* leads to an increased tiller angle due to asymmetric distribution of leaf internode cells and vascular bundles [8]. *BRANCHED1* (*BRC1*) is a crucial gene that regulates plant branching [9]. CKs positively regulate *BRC1* expression, promoting lateral bud growth and branching angle formation [10]. Additionally, isopentenyl transferases (IPTs) involved in CKs’ synthesis can inhibit *BRC1* expression, leading to a larger branching angle [11]. Strigolactones (SLs), key regulators of the branching angle, act as negative regulators of auxin and are critical in controlling lateral bud growth through the MORE AXILLARY GROWTH (MAX) and *WUSCHEL-RELATED HOMEOBOX4* (*WOX4*) pathways [12,13]. Abscisic acid (ABA) plays an important role in the dormancy and growth of lateral buds, with low levels of ABA promoting lateral bud growth [14]. Otherwise, *BRC* attenuates branching angle enlargement by promoting ABA synthesis [15]. Gibberellin (GA) is crucial for plant cell elongation [16]. In barley, auxin accumulation in the leaf pillow promotes an increase in GA1 content and increases the angle of the leaf pillow [17]. The GA-synthesising gene *GA3ox1* controls the development of plant branches and regulates endogenous hormone synthesis [18,19]. The IGT gene family, including *DEEPERROOTING 1* (*DRO1*), *TILLERANGLECONTROL 1* (*TAC1*), and *LAZY1*, regulates lateral bud growth and branching angle through auxin transport and gravitropic responses [12,20,21,22].

Branching angle significantly affects the shape and yield of the tea plant. Upright cultivars improve the integrity of buds and leaves during mechanical harvesting, making them ideal for breeding harvest efficient cultivars [23]. Research on branch development in tea plants has focused mainly on the effects of exogenous plant hormone spraying or mechanical damage to the branches. For example, *CsLAZY1* overexpression in *Arabidopsis* increased the branching angle under specific light and gravity conditions [24]. Meanwhile, 200 mg·L^−1^ 6-BA treatment reduced the number of lateral branches by 23.0% [25]. However, the mechanisms underlying branching angle formation in tea plants remain unclear. Given the role of the branching angle in mechanical harvesting, a deeper understanding of the endogenous factors driving branching angle formation is crucial [23].

In this study, three tea plant cultivars with distinct branching angles, representing the upright and draped types, were selected to investigate the molecular and hormonal mechanisms responsible for branching angle variation. By employing transcriptomics and profiling endogenous hormone level and Exogenous hormone treatment analyses, we aimed to uncover the key regulatory genes and hormonal pathways that govern branching angle formation in tea plants. The findings of this study provide valuable insights for breeding tea plant cultivars that are more suited to mechanised harvesting, thus improving agricultural efficiency and sustainability.

## 2. Results

### 2.1. Phenotypic Branching Angles of Different Tea Plant Cultivars

**Figure 1:**
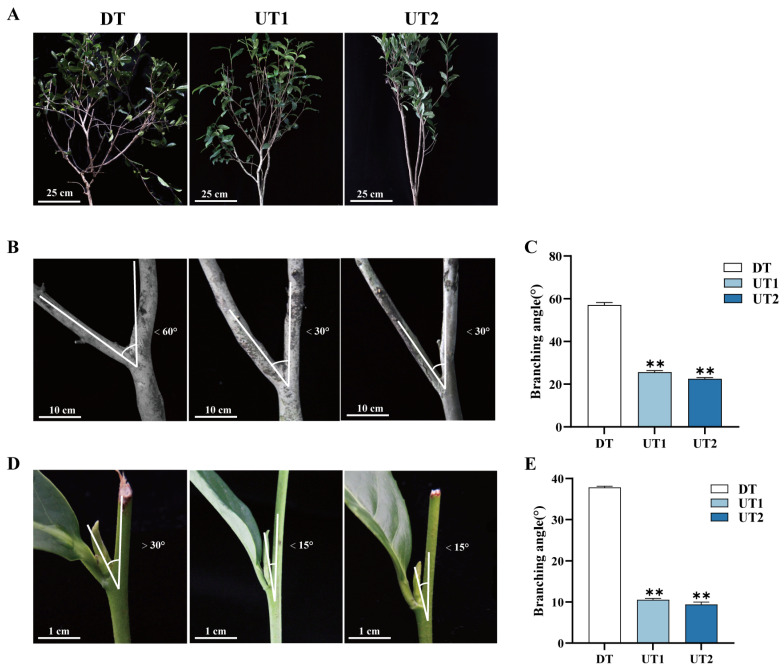
Plant architecture characteristics of the draped tea plant (DT) and the upright tea plant (UT). (**A**) Whole-plant phenotype. (**B**) Mature branches. (**C**) Branching angle of the mature branches. (**D**) New shoots. (**E**) Branching angles of new shoots. DT was designated as the control. Data are the means ± SE of fifteen biological replicates. Asterisks indicate significant differences (** *p* < 0.01), as determined by Student’s *t*-test.

Field observations of different tea plant cultivars revealed significant differences in their branching angles. The angle between the first-order branches of the upright cultivar UT1 and the vertical line of the ground is 25.65°, while for the upright cultivar UT2, it is 22.50°. In contrast, the first-order branches of the draped cultivar DT have an angle of 57.07° with respect to the vertical line of the ground (Figure 1A–C). Additionally, the crown shape of the upright tea plant is narrower, and has a branching angle of new shoots for UT1 of 10.55° and for UT2 of 9.40°, whereas the DT cultivar has a wider and looser crown shape, and has a new shoot branching angle of 37.84° (Figure 1D,E). Measurement of the branching angles of different tea plant cultivars showed that the branching angles of mature branches and shoots of the DT cultivar were significantly larger than those of the UT1 and UT2 cultivars.

### 2.2. Relationship Between Endogenous Hormones and the Branching Angle of Tea Plants

**Figure 2:**
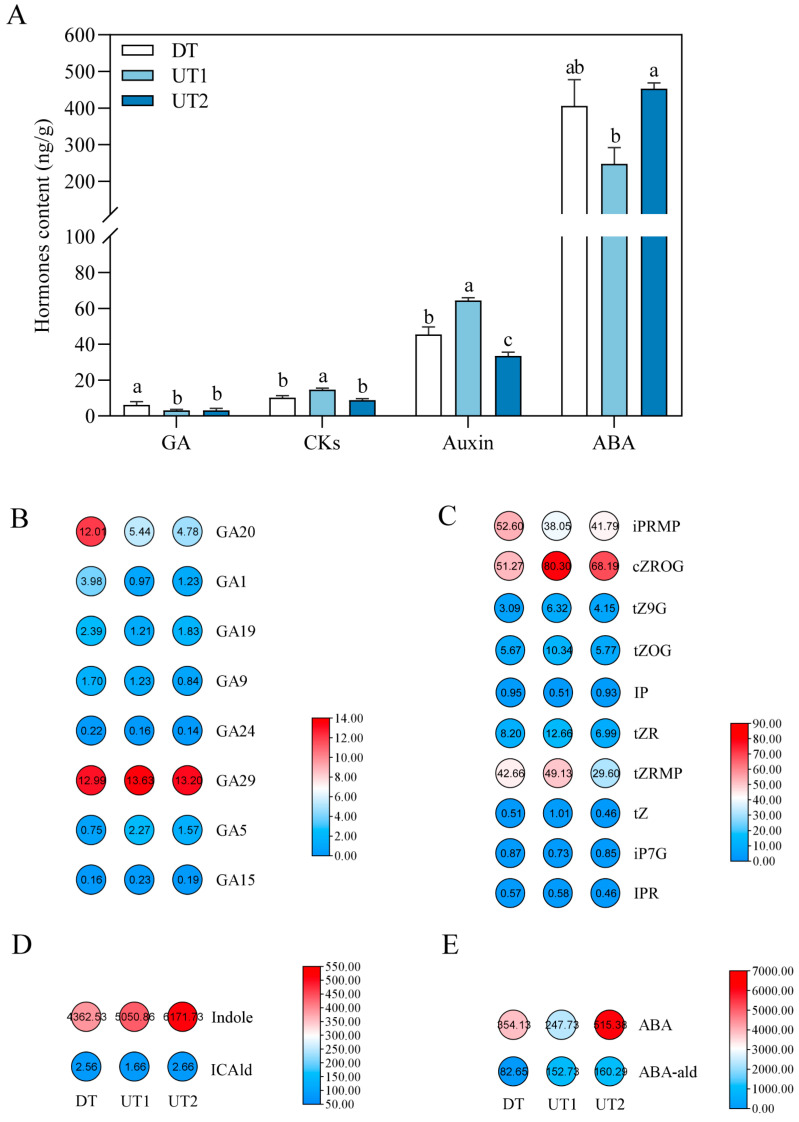
Analysis of plant hormones in UT and DT tea plant lateral buds. (**A**) The total amount of plant hormones in different tea plants. Data are the means ± SE of three independent biological replicates. Bars with different letters indicate significant differences (*p* < 0.05) as assessed by a one-way ANOVA, followed by Tukey’s HSD comparisons. (**B**) Heatmap of GA content. (**C**) Heatmap of CKs’ content. (**D**) Heatmap of Auxin content. (**E**) Heatmap of ABA content.

Endogenous hormones significantly influence branching development in plants. To clarify the regulatory role of endogenous hormones on branching development in tea plants, we determined the hormone content in the lateral buds of the UT and DT cultivars and found that the GA content in the UT cultivars was significantly lower than that in the DT cultivar (Figure 2A). The levels of GA components, such as GA5, GA29, and GA15 in the UT cultivars were higher than those in the DT cultivar, whereas the levels of GA1, GA24, GA19, GA20, and GA9 in the UT cultivars were lower than those in the DT cultivar (Figure 2B). The levels of CKs, such as cis-Zeatin-O-glucoside riboside (cZROG), trans-Zeatin-O-glucoside (tZOG), and trans-Zeaxin-9-glycoside (tZ9G), were higher in the UT cultivars compared with those in the DT cultivar (Figure 2C). Additionally, the content of the indole in the UT cultivars was higher than that in the DT cultivar (Figure 2D), and that of abscisic aldehyde (ABA-ald) in the UT cultivars was higher than that in the DT cultivar (Figure 2E). The content of GA was lower in the UT cultivars, whereas the content of most components of CKs, auxin, and ABA was higher in the UT cultivars. These findings suggest that GA may positively regulate the branching angle of tea plants and that CKs, auxin, and ABA may also be involved in the formation of the branching angle in tea plants.

### 2.3. GA3 Treatment Expands the Branching Angle in Tea Plant Shoots

**Figure 3:**
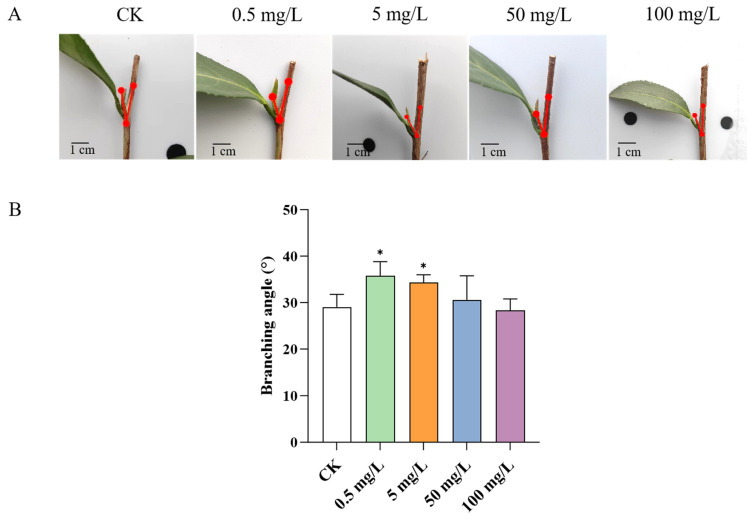
GA3 treatment Expands the branching angles in tea plant shoots. (**A**). Phenotypic of tea plant lateral buds treated with different concentrations of GA3. (**B**) The branching angles of tea plant lateral buds under GA3 treatment. Data are the means ± SE of fifteen biological replicates. Asterisks indicate significant differences (* *p* < 0.05), as determined by Student’s *t*-test.

GA promotes cell division and elongation in plants. To investigate the role of GA in the branching development of tea plants, we treated tea plants with GA3 at concentrations of 0.5 mg/L, 5 mg/L, 50 mg/L, and 100 mg/L, and measured the angle between the first lateral bud and the stem of the new shoots (Figure 3A). Our results revealed that the application of 0.5 mg/L GA3 led to a significant expansion in the branching angle of tea plant new shoots by 23.20%, while the 5 mg/L treatment resulted in an expansion of 18.24%, as illustrated in Figure 3B. Interestingly, the concentrations of 50 mg/L and 100 mg/L had no effect on the branching angle, suggesting that the lower GA concentrations might enhance the branching angle of tea plant lateral buds.

### 2.4. Comparative Transcriptome Analysis

**Figure 4:**
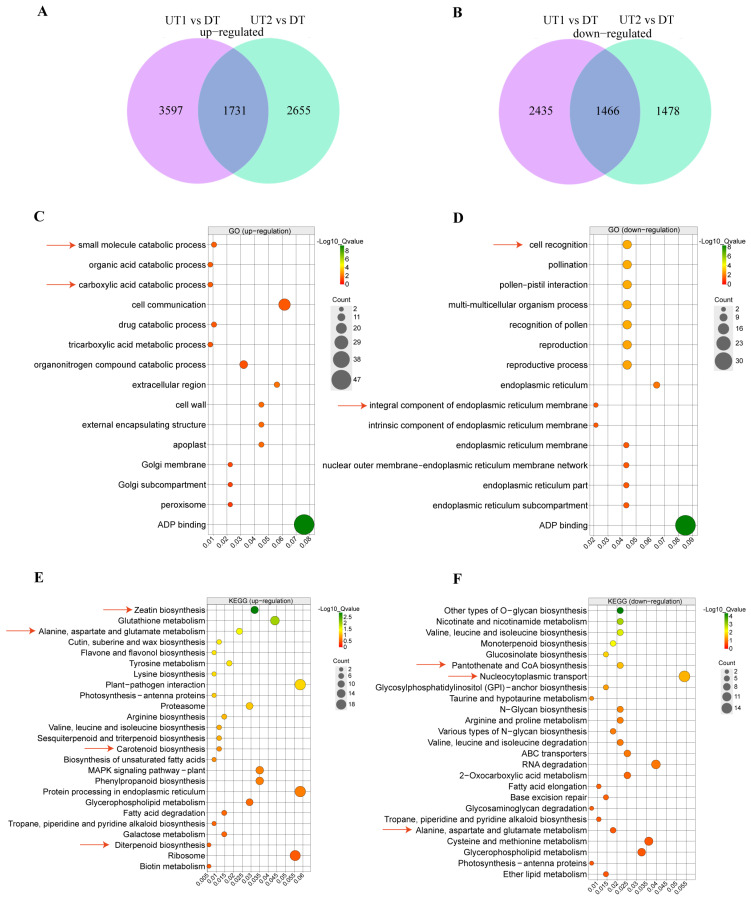
Venn diagram, GO, and KEGG analysis for DEGs. (**A**) Venn diagram showing up-regulated genes in the three cultivars lateral buds. (**B**) Venn diagram showing down-regulated genes in the three cultivars lateral buds. (**C**) GO term enrichment analysis for up-regulated genes (**D**) GO term enrichment analysis for down-regulated genes. (**E**) KEGG pathway analysis for up-regulated genes. (**F**) KEGG pathway analysis for down-regulated genes. The red arrows indicate the GO terms and KEGG related to the branching angle.

To elucidate the discrepancies in the molecular mechanisms of the branching angle formation between the UT and DT cultivars, RNA-seq analysis was conducted to generate transcriptome profiles. A total of 3627 differentially expressed genes (DEGs) were identified using a stringent statistical cut-off of |log_2_FoldChange| ≥ 1 and *p*-value < 0.05. Comparisons of the UT and DT cultivars revealed 1731 up-regulated genes (URGs) and 1466 down-regulated genes (DRGs) among the DEGs (Figure 4A,B, Table S1). The reproducibility of samples was confirmed by principal component analysis (Figure S1), demonstrating significant differences between the UT and DT cultivars. Based on the results of the Gene Ontology (GO) enrichment analysis, we observed significant enrichment (*q*-value < 0.05) of different key terms between the URGs and DRGs. Specifically, the significantly enriched terms associated with URGs were small-molecule catabolic and carboxylic acid catabolic processes (Figure 4C), whereas the significantly enriched terms associated with DRGs were cell recognition and integral components of the endoplasmic reticulum membrane (Figure 4D). Furthermore, a Kyoto Encyclopedia of Genes and Genomes (KEGG) enrichment analysis was conducted on the 1731 URGs (*q*-value < 0.05), which revealed significant enrichment of pathways related to the alanine, aspartate and glutamate metabolism, zeatin biosynthesis, carotenoid biosynthesis, and diterpenoid biosynthesis (Figure 4E, Table S2). KEGG enrichment analysis was also performed on the 1466 DRGs, which were significantly enriched in pathways such as the alanine, aspartate, and glutamate metabolism, pantothenic acid and CoA biosynthesis, and nucleocytoplasmic transport (Figure 4F, Table S3). The comparative analysis between GO and KEGG suggests that the development of branching angles in tea plant shoots is likely governed by cellular constituent, the synthesis and metabolism of small molecular compounds.

### 2.5. Transcription Factors Associated with Branching Development in Tea Plants

**Figure 5:**
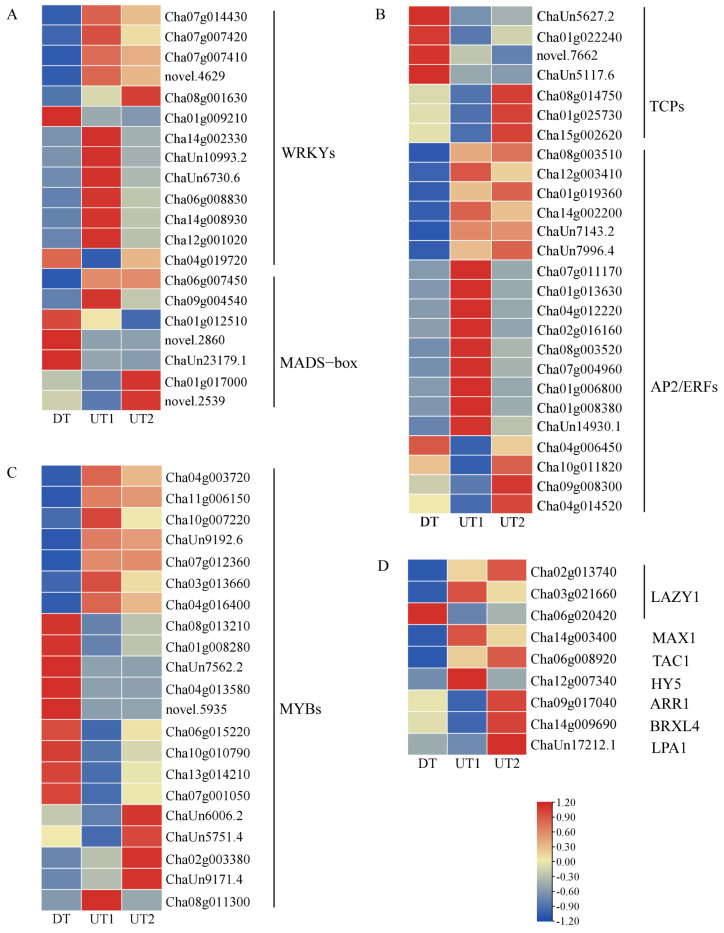
Identification of transcription factors associated with branching angle and DEGs affecting cell division in tea plants. (**A**) Heatmaps of WRKYs and MADS−box associated with transcription factors in tea plant lateral buds. (**B**) Heatmaps of TCPs and AP2/ERFs associated with transcription factors in tea plant lateral buds. (**C**) Heatmaps of MYBs associated with transcription factors in tea plant lateral buds. (**D**) Heatmap of DEGs associated with cell division in tea plant lateral buds.

Transcription factors (TFs) play a pivotal role in orchestrating plant growth. In the lateral buds of tea plants, 1101 TFs were identified, spanning 66 families (Figure S2A). A total of 67 differentially expressed TFs were significantly enriched in tea plant lateral buds (*p*-value < 0.05), which were related to plant growth development and belonged to the WRKYs, AP2/ERFs, MYBs, TCPs, and MADS-box gene families (Figure S2B). Specifically, the expression of WRKYs (*Cha07g014430*, *Cha07g007420*, *Cha07g007410*, and *Cha08g001630)*, AP2/ERFs *(Cha08g003510*, *Cha12g003410*, *Cha01g019360*, *Cha14g002200*, *ChaUn7143.2*, and *ChaUn7996.4*), MYBs (*Cha04g003720*, *Cha11g006150*, *Cha10g007220*, *ChaUn9192.6*, *Cha07g012360*, *Cha03g013660*, and *Cha04g016400*) was up-regulated in the UT cultivars, whilst the expression of MADS-box (*Cha01g012510* and *ChaUn23179.1*) and TCPs (*ChaUn5627.2*, *Cha01g022240*, and *ChaUn5117.6*) was down-regulated in the UT cultivars. Moreover, the expression of *LAZY1* (*Cha02g013740* and *Cha03g021660*), *TAC1* (*Cha06g008920*), and *MAX1* (*Cha14g003400*) was up-regulated in the UT cultivars (Figure 5A–D). These results indicated that the expression levels of WRKYs, AP2/ERFs, MYBs, *LAZY1*, *TAC1*, and *MAX1* were higher in the UT cultivars compared with those in the DT cultivar during the formation of the branching angle in tea plants, which may result in the smaller branching angle. Meanwhile, the expression of MADS-box and TCPs was down-regulated in the UT cultivars, which may be regulated by other genes, thereby promoting an increase in the branching angle.

### 2.6. Combined Analysis of Endogenous Phytohormones and DEGs Related to Plant Hormone Biosynthesis, Metabolism, and Signal Transduction Pathways in the Tea Plant Lateral Bud

To clarify the regulatory mechanisms of the branching angle by hormone-related genes, we identified 24 hormone-related DEGs in the transcriptome, which were mainly enriched in CKs, auxin, GA, and ABA-related pathways (|log_2_FoldChange| ≥ 1 and *p*-value < 0.05, Figure S3, Table S4).

**Figure 6:**
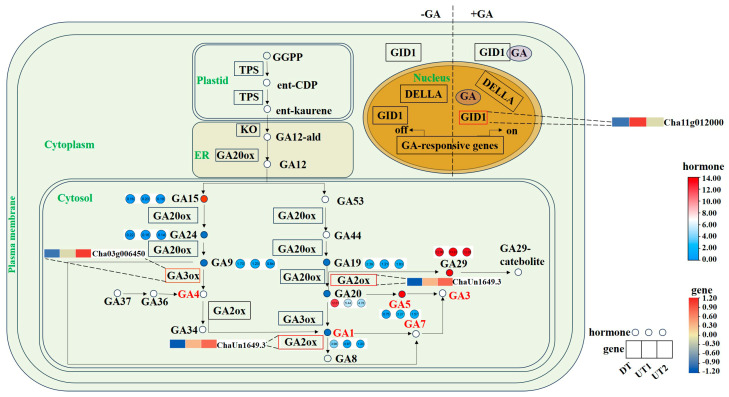
GA biosynthesis, metabolism, and signal transduction pathways. The green font represents organelle names, the red font represents active hormones, the circle represents plant hormones, the rectangle represents genes, the red circle represents high expression of plant hormones in the UT1 and UT2 cultivars, and the blue circle represents low expression of plant hormones in the UT1 and UT2 cultivars.

In the GA biosynthesis pathway, the precursor GGPP is first produced in plastids, followed by the synthesis of GA12 in the endoplasmic reticulum. GA12 is an important precursor for the synthesis of active GA and may be regulated by gibberellin oxidase during the synthesis of GA1 and GA4 (Figure 6). Our findings revealed that *Cha03g006450 (GA3ox1)* was up-regulated in UT cultivars. Additionally, we observed that the levels of GA1 and GA20 were lower in UT cultivars compared to the DT cultivar. *ChaUn1649.3* (*GA2ox1*) was up-regulated in the UT cultivars, may result in lower levels of GA1 in the UT cultivars compared with those in the DT cultivar. Furthermore, we found that the GA5 content in the UT cultivars was higher than that in the DT cultivar. This indicated that hormones and genes related to GA synthesis play important roles in the formation of the branching angles in tea plants.

**Figure 7:**
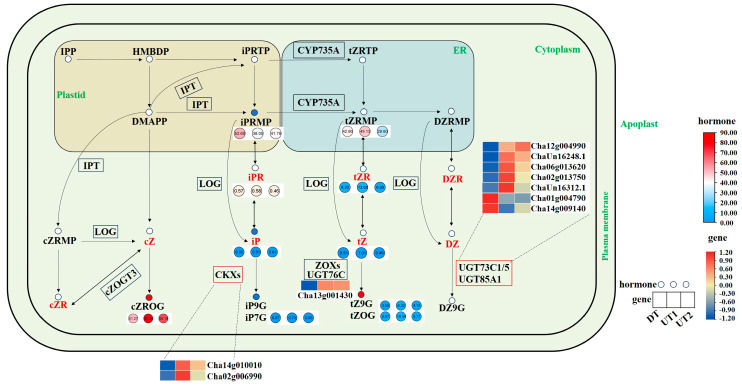
Cytokinin biosynthesis, metabolism, and signal transduction pathways. The green font represents organelle names, the red font represents active hormones, the circle represents plant hormones, the rectangle represents genes, the red circle represents high expression of plant hormones in the UT1 and UT2 cultivars, and the blue circle represents low expression of plant hormones in the UT1 and UT2 cultivars.

In the CKs’ synthesis pathway, the N6-Isopentenyl-Adenosine-5′-Monophosphate (iPRMP) levels were reduced during the synthesis of N6-isopentenyladenine (iP) and trans-Zeatin (tZ), the iP level in UT cultivars was lower than in DT cultivar, while there was no significant difference in the tZ levels between the UT and DT cultivars (Figure 7). During plant development, iP is transported from the bud to the root. During iP metabolism to N6-Isopentenyl-adenine-7-glucoside (iP7G) and N6-Isopentenyl-adenine-9-glucoside (iP9G), iP is regulated by *CKX* genes. *Cha14g010010* (*CKX5*) was up-regulated by 3.60- and 3.12-fold in the UT cultivars. Additionally, 7 glycosyltransferase genes (*UGTs*) were significantly enriched during the degradation of DZ to DZ9G, and the expression of *Cha06g013620* (*UGT73C3.1*) was 4.46- and 3.80-fold higher in the UT1 and UT2 cultivars, respectively, compared with that in the DT cultivar. Therefore, CKs and their degradation-related genes play key roles in regulating branching angle formation in tea plants.

Auxin synthesis and metabolism occur within the cytoplasm, whereas auxin signal transduction occurs in the nucleus, cytoplasm, and apoplasm [16]. The process of auxin decomposition to Indole-3-acetyl glutamic acid (IAA-Glu) and Indole-3-acetyl-aspartate (IAA-Asp) may be regulated by *GH3*, which exhibited up-regulated in the UT cultivars (Figure S4). Additionally, *Cha05g013380* (*GH3.6.1*) was up-regulated by 1.56- and 1.16-fold in the UT1 and UT2 cultivars compared with the DT cultivar. Notably, IAA is achieved through *AUX/LAX* response to auxin signals and specific binding to IAA, whereas *LAX3* was up-regulated in the UT cultivars. *LAZY1* regulates the asymmetric distribution of auxin within plants to control their branching angle. In the present study, the expression of *Cha06g020420* (*LAZY1*) was 1.52- and 1.08-fold lower in the UT1 and UT2 cultivars than in the DT cultivar, respectively. The expression of *Cha06g008920* (*TAC1*), a negative regulator of *LAZY1*, was 3.00- and 3.53-fold higher in the UT1 and UT2 cultivars, respectively, compared with that in the DT cultivar. During the biosynthesis and metabolism of ABA, *Cha12g005490 (CYP707A2)* was highly expressed in the UT cultivars, but there was no significant difference in the ABA content between the UT and DT cultivars (Figure S5). These results indicate that *LAZY1* and *TAC1* interact to regulate auxin signal transduction in tea plant lateral buds, thereby affecting the expression of auxin-signal-transduction-related genes. During ABA signal transduction, the expression of the ABA receptor *Cha10g003740* (*PYL4*) was up-regulated in UT cultivars, promoting ABA signal transmission, indicating that the PYL4 receptor is more sensitive to ABA. The above results show that GA, CKs, auxin, ABA, and their genes play important roles in the formation of the branching angle in tea plants.

### 2.7. Transcriptome Validation Using qRT-PCR

**Figure 8:**
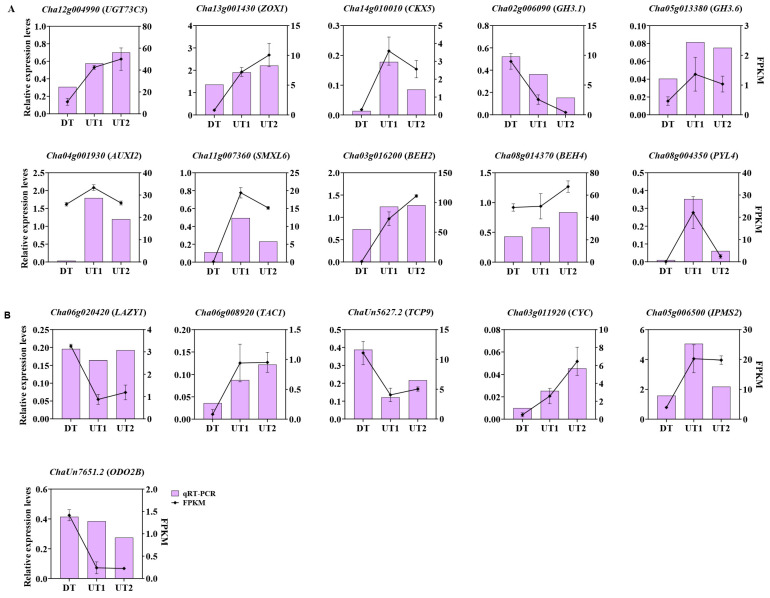
Expression of differentially expressed genes in tea plant lateral buds. (**A**) Genes associated with plant hormones. (**B**) Genes associated with cell division and growth in the lateral bud of tea plant.

To verify the accuracy and reliability of the RNA-Seq data, we screened 16 genes related to plant hormones (Figure 8A) and growth development (Figure 8B) via qRT-PCR analysis in the lateral buds. The expression patterns of 16 genes in the lateral buds demonstrated by the RNA-Seq and qRT-PCR results were consistent. Therefore, the RNA-Seq data were considered reliable.

## 3. Discussion

Many factors affect the formation of tea plant branching angles [26]. Tea plants are among the most popular and economically important plants worldwide. The branching angle of tea plants is a significant indicator of plant yield and suitability for machine harvesting. Plant cultivars with small branching angles suitable for mechanical harvesting can effectively improve harvesting efficiency. Plant hormones and their related genes significantly affect the formation of plant branching angles [27]. In this study, the tea plant cultivars with significantly different branching angles were selected as research subjects. Through combined analysis of the transcriptome and plant hormone levels, we found that the branching angle of tea plants is not regulated by a single factor, such as hormone content, but by a variety of hormones and their related genes.

### 3.1. Plant Hormones Are the Key Factors Affecting the Formation of the Tea Plant Branching Angles

Plant hormones play vital roles in the growth of new shoots [28]. Auxin affects the expression of gibberellin oxidases. For example, auxin promotes the expression of *GA20ox* genes and *GA3ox* genes in pea, while inhibiting the expression of *GA2ox* genes to regulate plant branching development [29]. In rice, the uneven distribution of auxin induced by gravity leads to GA 3β hydroxylase-induced asymmetric expression of the *OsGA3ox1* at the base of the leaf sheath [30]. In *Arabidopsis* and rice, *GA2ox* genes overexpression results in decreased GA content, leading to increased plant branching/tillering, which may be the result of the interaction between the GA and the auxin efflux carrier PIN [31]. In this study, it was found that the content of GA endogenous hormones in the UT cultivars was lower than that in the DT cultivar, and *GA3ox1* and *GA2ox1* were involved in the formation of branching angles in tea plants. At the same time, exogenous GA3 treatment could expand the branching angle of tea plant shoots. It can be seen that GA positively regulates the branching angle of tea plants.

Auxin inhibits the expression of CKs’ synthesis genes and up-regulates the expression of CKs’ degradation-related genes [32]. Auxin, SLs, and CKs are thought to regulate the growth of lateral buds by controlling the outward transport of auxin [33]. Auxin and SLs have opposite effects to CKs. Auxin regulates tillering in rice by controlling the expression of *OsIPT* [34]. In addition, auxin regulates CKs’ degradation through *CKX* genes, thereby controlling branching [35]. IP is regulated by *CKX* genes during bud orientation and root transport [4]. In this study, the expression of *CKX5* and *CKX6*, which are involved in the iP metabolism pathway, was up-regulated in the UT cultivars. No related genes were enriched in the CKs’ synthesis and signal transduction pathways, consistent with the results that showed no regularity in the active CKs’ contents between the UT and DT cultivars. Previous research on the sprouting mechanism of lateral buds in tea plants identified *CKX* as a key factor [36]; these results suggest that *CKX* genes may play a key role in the decomposition of iP. No significant difference in active CKs’ contents was observed between the UT and DT cultivars, which may have been caused by the regulation of auxin and its associated genes.

LAX is an auxin transport protein that negatively regulates auxin signal [37]. In this study, *LAX3* was only up-regulated in the UT cultivars, indicating that *LAX* plays a strong role in regulating auxin influx and maintaining auxin accumulation in the lateral buds, which is consistent with the roles of *SILAX3* and *AtLAX3* in tomato and *Arabidopsis*, respectively [38,39]. As an important gene regulating auxin transport, the expression level of *LAZY1* in the UT cultivars was significantly lower than that in the DT cultivar, consistent with previous results in other crops, which showed that its overexpression leads to an increased tiller angle [2]. *TAC1,* the main gene regulating lateral shoot orientation in *Arabidopsis*, has an opposite regulatory effect to that of *LAZY1* [20]. In the UT cultivars, the *TAC1* was up-regulated. These results suggest that *LAZY1* and *TAC1* may regulate the formation of tea plant branching angles; that is, *LAZY1* and *TAC1* regulate the transport of IAA. *LAX3* further facilitates the transport of IAA from other tissues to lateral buds through the membrane, thereby regulating the formation of tea plant branching angles. However, the specific regulatory mechanism requires further exploration.

During the germination of lateral buds, auxin inhibits CKs while promoting SLs. Concurrently, SLs and CKs stimulate ABA synthesis by inhibiting *BRC1*, thereby suppressing the growth of lateral buds [34]. ABA act as an inhibitor of plant growth, along with SLs, which regulate the lateral bud growth [40]. In *Arabidopsis*, ABA can bind to auxin and exert its inhibitory effect on axillary buds during certain stages of the cell cycle [41]. In the present study, the expression of the *PYL4* was up-regulated in the UT cultivars, but the ABA content did not differ significantly between the UT and DT cultivars. This may be attributed to the interaction between auxin and ABA in the regulation of cell division and growth. Therefore, we deduced that GA positively regulated the branching angle of tea plants, with CKs, auxin, and ABA involved.

### 3.2. Transcription Factors Regulate the Formation of the Tea Plant Branching Angle

During plant morphogenesis, the selective expression of genes leads to phenotypic differentiation, and TFs play important regulatory roles in these processes [28]. The *AtTCP9* in *Arabidopsis* regulates the division and growth of leaf cells and may affect lateral branch development [42]. A total of 37 *TCP* genes have been identified in tea plants [43]. Among these, we found that *ChaUn5117.6* (*TCP9*) was significantly down-regulated in the UT cultivars, consistent with the results obtained in previous studies. Therefore, we hypothesised that *TCP9* is involved in the formation of branching angles in tea plants by regulating cell wall stretching. MYBs regulate plant growth. In the present study, most *MYB* genes were up-regulated in the UT cultivars, which contradicts the results in rice, where *OsMYB48* promoted cell elongation and tillering [44]. This variation may be attributed to the differential expression of *MYB* in different cultivars. The AP2/ERF family is one of the main gene families that regulates plant growth. In chrysanthemum, *ERF53* overexpression enhances cell division, leading to an increase in rosette branching [45]. Therefore, TFs such as MYBs, WRKYs, AP2/ERFs, TCPs, and MADS-box, as well as plant-growth-related genes, including *LAZY1, TAC1*, and *MAX1*, are closely related to the growth of lateral buds in tea plants.

The limitations of this study lie in the different genetic backgrounds of the experimental materials. Even with strict control over the management and sampling conditions of the experimental materials, the results of plant hormone profiles of similar tree-type tea plants may still show significant differences due to many factors, such as genetic complexity and the intricacy of hormones. In the future, tea plant populations with the same genetic background should be used as experimental materials and the consistency of the growth environment should be maintained to overcome the limitations that arise from genetic differences, which may prevent experimental results from meeting expectations.

## 4. Materials and Methods

### 4.1. Plant Material and Sample Collection

According to the standard NY/T 2422–2013 (http://www.nybkjfzzx.cn/RESOURCES/ZBFiles/20155251570677.pdf, accessed on 14 December 2024), tea plant cultivars are classified based on the angle between the first-order branches and the vertical line of the ground as follows: cultivars with an angle of less than 30° are referred to as upright cultivars, cultivars with an angle between 30° and 50° are called semi-draping cultivars, and cultivars with an angle greater than 50° are known as draped cultivars.

The experimental plants were cultivated in a germplasm resource garden located at the Tea Research Institute of the Chinese Academy of Agricultural Sciences (Hangzhou, China; 30.18° N, 120.09° E). Two upright tea plant cultivars (UT) of ‘CT2009-0025’ (upright tea 1 (UT1)), ‘CT2013-0114’ (upright tea 2 (UT2)), and ‘CT2009-0160’, which is a draped tea plant cultivar (DT), were measured and the lateral buds were sampled. These plants were subjected to decapitation treatment. When the first lateral bud reached the swollen stage (about 2 weeks after bud formation), the branching angles of the three tea cultivars were measured, and samples of the lateral buds were collected. Three biological replicates were used for each tea cultivar, with at least 30 lateral buds per replicate. The samples were meticulously divided into three portions: the first was allocated for transcriptome sequencing, the second for assessing plant hormone levels, and the third was promptly plunged into liquid nitrogen before being stored at −80 °C for future analysis.

The technique for measuring the branching angle of the tea plant’s new shoots involved securing the shoot branches, which included both the stem and lateral buds, within the crop angle measuring device (TPZW-J-1, Hangzhou Top Instrument Co., Ltd., Hangzhou, China). Following this, the branches were scanned to determine the angle formed between the two tangents extending from the stem and the lateral bud.

### 4.2. RNA Extraction, Library Construction, and Sequencing

A total of 400 ng total RNA was extracted from the lateral buds of different tea plant cultivars and the integrity and purity of the extracted RNA were assessed with Fu’s method; 3 replicates were set for each tea plant cultivar [46]. A sequencing library was generated using the NEBNext^®^ UltraTM RNA Library Prep Kit for Lumina^®^ (New England Biolabs, Ipswich, MA, USA), following the manufacturer’s instructions. The sequencing and transcriptome data were performed by Novogene Company (Beijing, China). In this study, the whole genome of Longjing 43 was selected as the reference genome, and the reference genome and annotation files were downloaded from the National Genomics Data Center (https://ngdc.cncb.ac.cn/search/?dbld=gwh&q=GWHACFB00000000 (accessed on 31 July 2023)) [47]. Clean reads were compared with the reference genome using the HISAT2 software (v 2.0.5), which provided efficient and accurate mapping of the reads to specific genomic locations [48].

### 4.3. Transcriptomic Data Analysis

The DESeq2 package in R software (v 4.1.0) was used to analyse DEGs between UT1 vs. DT and UT2 vs. DT [49]. A negative binomial distribution was used to model the count data and perform the hypothesis test, with *p*-values calculated for each gene. To control for multiple testing, the *p*-values were corrected using the Benjamini–Hochberg method to control the False Discovery Rate (FDR). In the pairwise comparison, DEGs with |log_2_FoldChange| ≥ 1 and *p*-value < 0.05 were clustered. The Novogene platform (https://magic.novogene.com/customer/main#/homeNew (accessed on 7 August 2023)) was used to construct a Venn diagram, Heatmaps. Column charts, Gene Ontology (GO), and KEGG enrichment analyses were performed using GraphPad Prism 8 and a website (https://www.chiplot.online/ (accessed on 9 July 2024)) [50].

### 4.4. Verification of RNA-Seq Results Using qRT-PCR

RNA was reverse-transcribed into cDNA using a PrimeScript RT kit (RR047A, Takara, Dalian, China), and cDNA was diluted 10-fold. Gene-specific primers were designed using Oligo7, the primer sequences are listed in Table S5, and *CsPTB* was selected as the reference gene [51]. The genes were subjected to qRT-PCR using a SYBR Green I Master kit (Roche, Basel, Switzerland), and the relative expression level of each gene was calculated using a 2^−∆Ct^ method.

### 4.5. Plant Endogenous Hormone Measurement in the Lateral Bud

CKs, ABA, and auxin were detected following the approach described by Zhou et al. [52]. Plant hormone content was measured using the OTRAP6500 + LC-MS/MS platform (http://www.metware.cn/ (accessed on 10 August 2023)). All measurements had three biological replicates.

The determination of GA content involved weighing 50 mg of plant sample into a 2 mL plastic microtube and extraction with a mixture of methanol/water/formic acid (15:4:1, *v*/*v*/*v*). Then, the extraction solution was added with 10 μL of internal standard solution (10 ng/mL) for quantification. Subsequent steps for GA content measurement were performed on the QTRAP 6500 + LC-MS/MS to analyse the hormone content in the lateral bud samples.

### 4.6. Exogenous Hormone Treatment

On 22 April 2024, an experiment was conducted using the upright tea plant Zhong Cha 108 (ZC108) cultivated at the Tea Research Institute of the Chinese Academy of Agricultural Sciences in Hangzhou, China (30.18° N, 120.09° E). The tea plants were subjected to light pruning, which involved trimming 15–20 cm above the previous cut. On the fifth day after pruning, exogenous gibberellin (GA3) was applied to the test materials via full-tree spraying at 4:00 PM, with the spray volume adjusted to ensure that the leaves dripped. The concentrations used for the application were 0.5 mg/L, 5 mg/L, 50 mg/L, and 100 mg/L, with an equal volume of pure water serving as the control. GA3 was dissolved in anhydrous ethanol. Each treatment has three biological replicates. On the 20th day after application, when the first lateral bud of the tea plant shoot had reached the swelling stage, the crop-angle-measuring device (TPZW-J-1, Hangzhou Top Instrument Co., Ltd., Hangzhou, China) was employed to measure the branch angle between the tea plant shoot stem and the first lateral bud under the different GA3 concentrations, with fifteen replicates taken for each treatment.

### 4.7. Statistics Analysis

All data were analysed in SPSS Statistics v. 26 (IBM Corp., Armonk, NY, USA) using either Student’s *t*-test or one-way ANOVA. For pairwise comparisons, post hoc Tukey’s HSD test was performed to correct for multiple comparisons. The results were represented as the mean ± SE. Statistical significance was set at *p* < 0.05. Graphing and image analysis were carried out using GraphPad Prism 8 software (GraphPad software, La Jolla, CA, USA), and Adobe Illustrator 2021.

## 5. Conclusions

**Figure 9:**
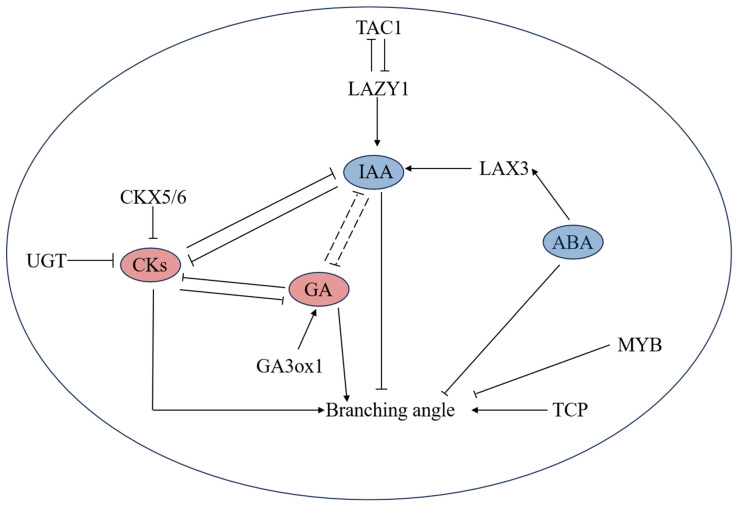
Mechanism model of tea plant branching angle formation. The solid line represents the direct effect, the dotted line indicates the indirect effect, the arrow indicates the positive regulation, and the thick line represents the negative regulation. The red color represents a hormone that positively regulates the branching angle, the blue color represents a hormone that negatively regulates the branching angle. Abbreviations: ABA, Abscisic acid; IAA, Indole-3-acetic acid; GA, Gibberellin; CKs, Cytokinins; CKX5/6, Cytokinin oxidase/dehydrogenase 5/6; UGT, Uridine diphosphate glycosyltransferase; GA3ox1, GA3-oxidase 1; ERF, Ethylene-responsive element binding factors; TAC1, TILLER ANGLE CONTROL 1; LAX3, AUXIN3/LIKE-AUX3; TCP, TEOSINTE BRANCHED1/CYCLOIDEA/PROLIFERATING CELL FACTORS.

This study explored the mechanism underlying the branching angle of new shoots and identified key factors affecting the development of lateral buds in tea plant cultivars with different plant structures. Through transcriptome and plant hormone analyses, key genes and metabolites involved in tea plant lateral bud development were identified, and a comprehensive regulatory pathway for the formation of tea plant branching angles was proposed (Figure 9). This pathway is intricately linked to various factors, with plant hormones emerging as key factors in branch development. Genes related to hormone synthesis, metabolism, and signal transduction participate in this complex regulatory process. Overall, this study provides insights into the complex mechanisms of tea plant branching angle formation and offers a reference for selecting tea plant cultivars suitable for mechanical harvesting.

## Data Availability

All data generated or analysed during this study are included in the published article and Supplementary Files.

## Supplementary Materials

The following supporting information can be downloaded at: https://www.mdpi.com/article/10.3390/ijms26020604/s1.
