# Supplementary figures and images for: Transcriptomics and Plant Hormone Analysis Reveal the Mechanism of Branching Angle Formation in Tea Plants (Camellia sinensis)
Source: Int J Mol Sci. 2025 Jan 13;26(2):604. doi: 10.3390/ijms26020604 (PMC11765265; doi:10.3390/ijms26020604)

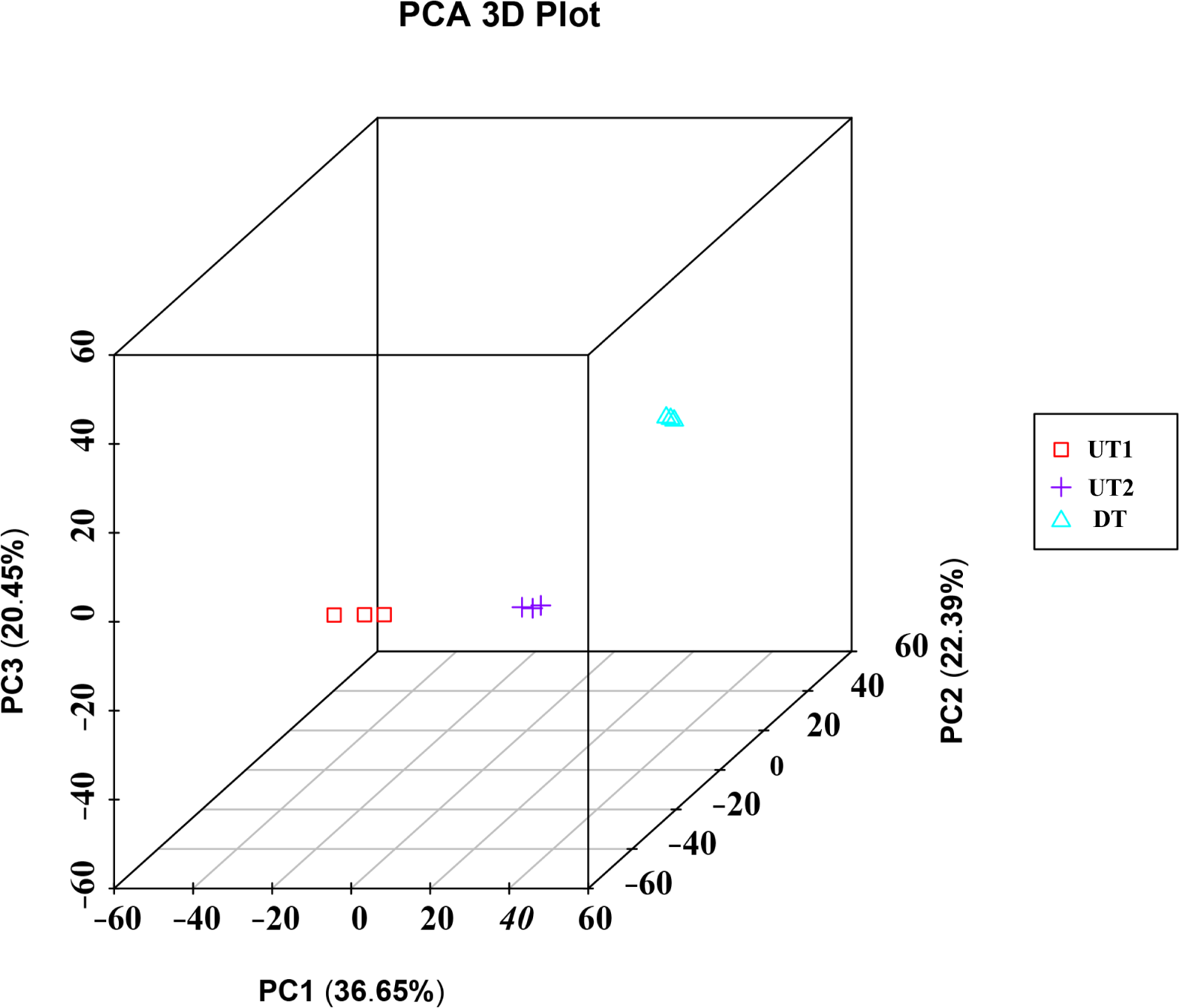

Supplement: Supplementary file 1 [file ijms-26-00604-s001.zip › Figure S1.tif]

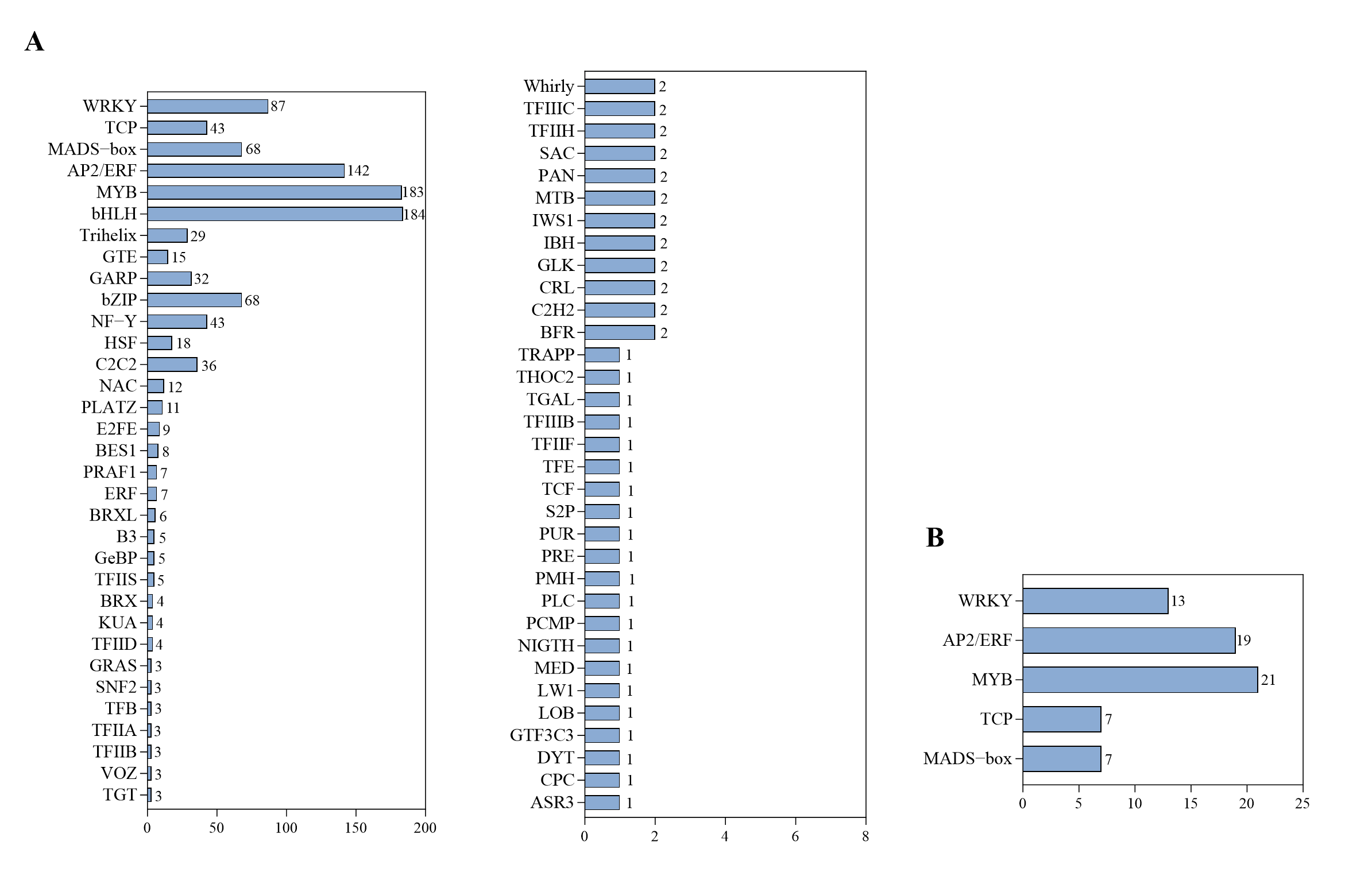

Supplement: Supplementary file 1 [file ijms-26-00604-s001.zip › Figure S2.tif]

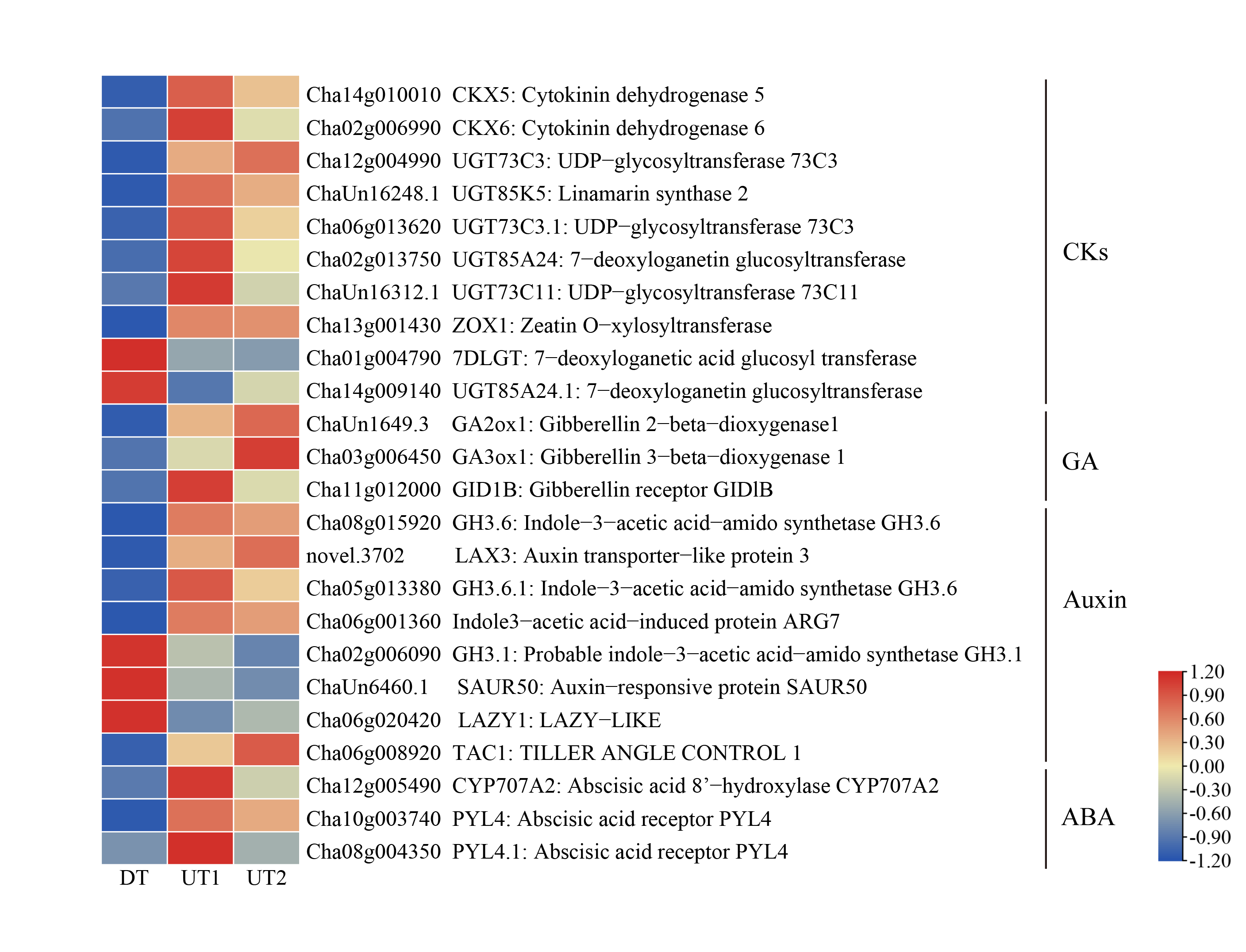

Supplement: Supplementary file 1 [file ijms-26-00604-s001.zip › Figure S3.tif]

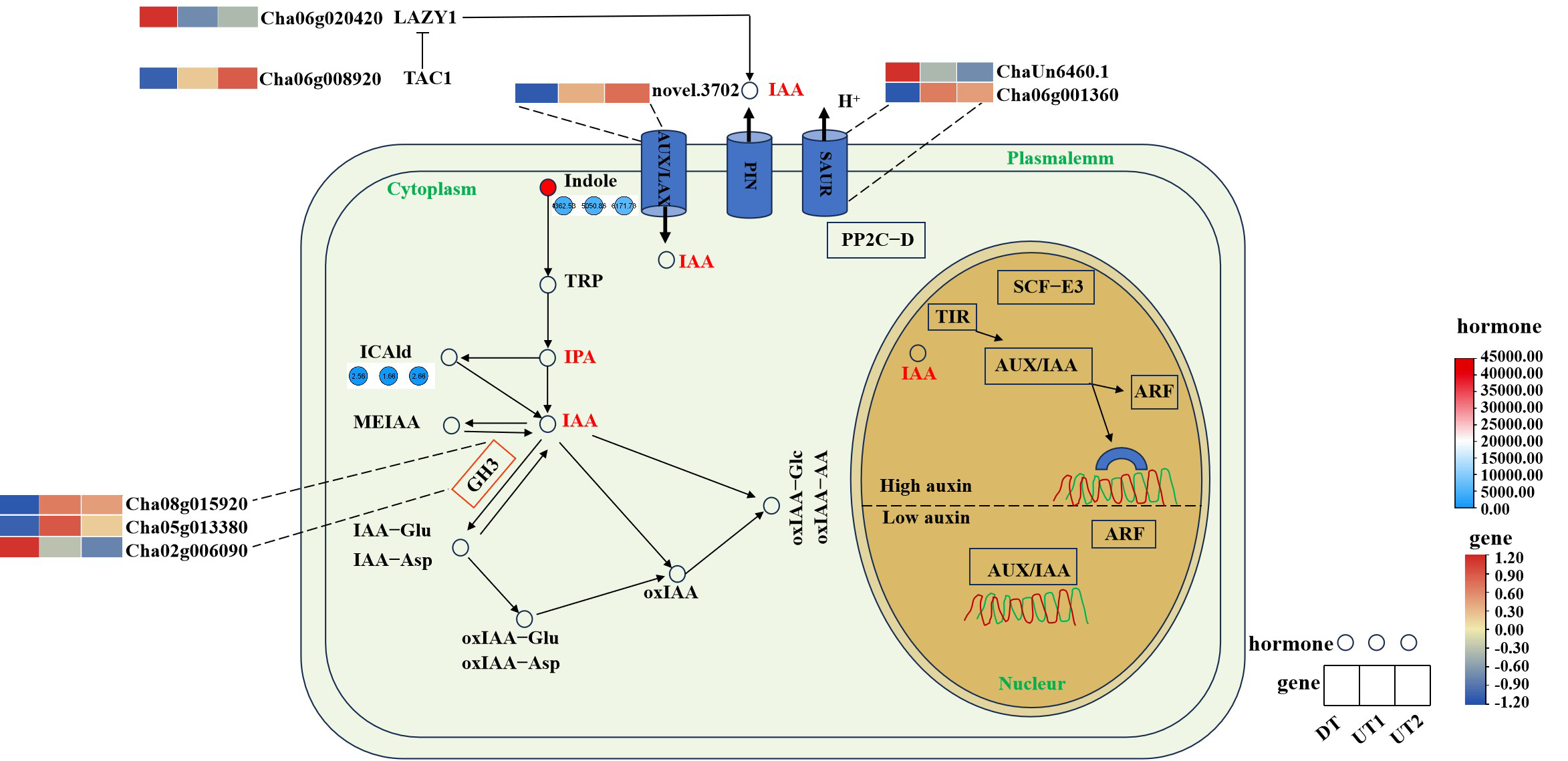

Supplement: Supplementary file 1 [file ijms-26-00604-s001.zip › Figure S4.tif]

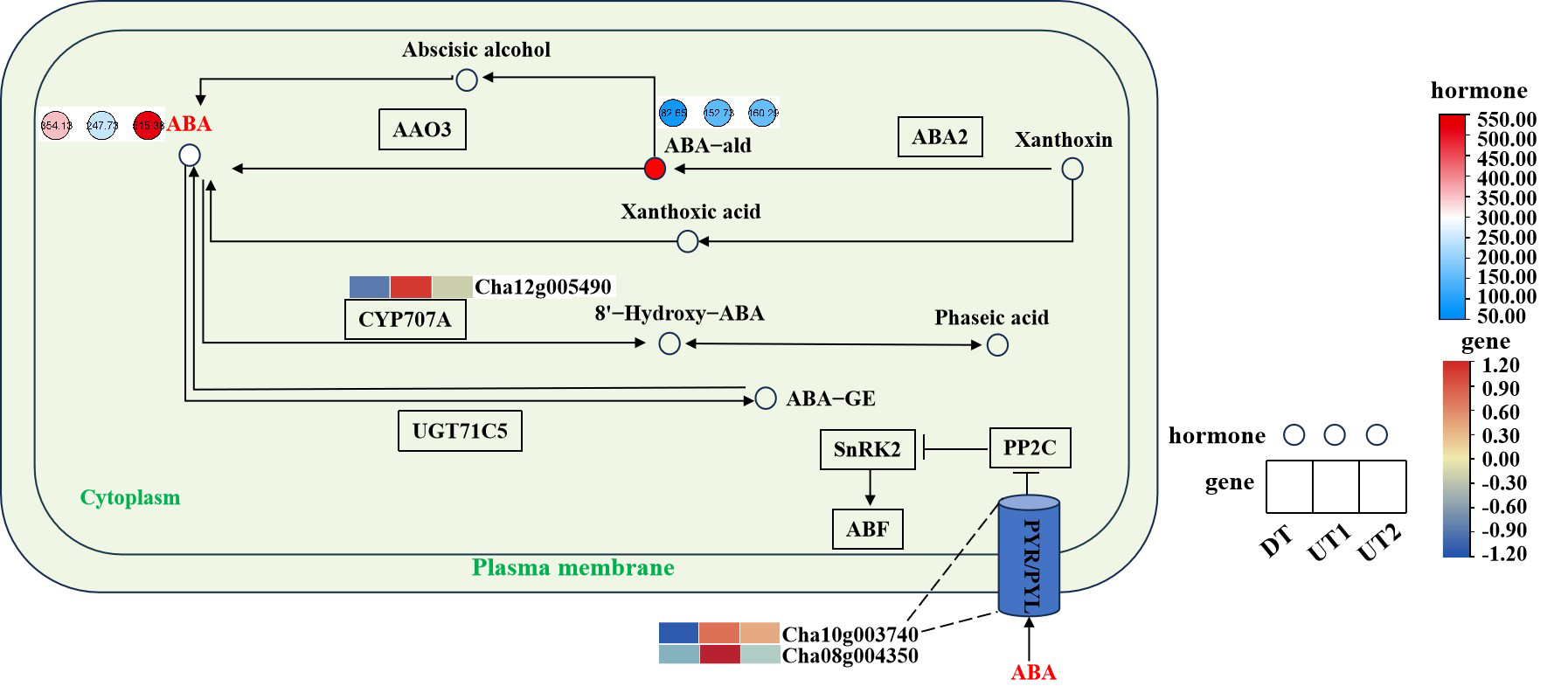

Supplement: Supplementary file 1 [file ijms-26-00604-s001.zip › Figure S5.tif]
